# Contraception needs and pregnancy termination in sub-Saharan Africa: a multilevel analysis of demographic and health survey data

**DOI:** 10.1186/s12978-021-01227-3

**Published:** 2021-08-28

**Authors:** Kenneth Setorwu Adde, Kwamena Sekyi Dickson, Edward Kwabena Ameyaw, Joshua Amo-Adjei

**Affiliations:** 1 Department of Population and Health, College of Humanities and Legal Studies, University of Cape Coast, Cape Coast, Ghana; 2 The Australian Centre for Public and Population Health Research, Faculty of Health, University of Technology Sydney, Sydney, NSW Australia

**Keywords:** Unmet/met need, Contraception, Unsafe abortion, Pregnancy termination

## Abstract

**Background:**

Women in sub-Saharan Africa (SSA) have a higher risk of unintended pregnancies that are more likely to be terminated, most of which are unsafe with associated complications. Unmet need for contraception is highest in SSA and exceeds the global average. This study investigates the association between unmet/met need for contraception and pregnancy termination SSA.

**Methods:**

We used pooled data from Demographic and Health Surveys conducted from January 2010 to December 2018 in 32 countries in SSA. Our study involved 265,505 women with diverse contraception needs and with complete data on all variables of interest. Multilevel logistic regression at 95% CI was used to investigate the association between individual and community level factors and pregnancy termination.

**Results:**

We found an overall pregnancy termination rate of 16.27% ranging from 9.13% in Namibia to 38.68% in Gabon. Intriguingly, women with a met need for contraception were more likely to terminate a pregnancy [aOR = 1.11; 95% CI 1.07–1.96] than women with unmet needs. Women with secondary education were more likely to terminate a pregnancy as compared to those without education [aOR = 1.23; 95% CI 1.19–1.27]. With regards to age, we observed that every additional age increases the likelihood of terminating a pregnancy. At the contextual level, the women with female household heads were less likely to terminate a pregnancy [aOR = 0.95; 95% CI 0.92–0.97]. The least socio-economically disadvantaged women were less likely to terminate a pregnancy compared to the moderately and most socio-economically disadvantaged women.

**Conclusions:**

Our study contributes towards the discussion on unmet/met need for contraception and pregnancy termination across SSA. Women with met need for contraception have higher odds of terminating a pregnancy. The underlying cause of this we argued could be poor adherence to the protocols of contraceptives or the reluctance of women to utilise contraceptives after experiencing a failure. Governments of SSA and non-governmental organisations need to take pragmatic steps to increase met needs for contraception and also utilise mass media to encourage women to adhere to the prescription of contraceptives in order to reduce the incidence of unplanned pregnancies and unsafe abortions.

## Plain language summary

Women in sub-Saharan Africa (SSA) have a higher risk of unintended pregnancies that are more likely to be terminated, most of which are unsafe with associated complications. Unmet need for contraception is highest in SSA and exceeds the global average. This study investigates the association between unmet/met need for contraception and pregnancy termination SSA. We used pooled data from Demographic and Health Surveys conducted from January 2010 to December 2018 in 32 countries in SSA. Our study involved 265,505 women with diverse contraception needs and with complete data on all variables of interest. We found an overall pregnancy termination rate of 16.27% ranging from 9.13% in Namibia to 38.68% in Gabon. Met need of contraception, education, sex of household head, and socio-economic disadvantage of women had a significant association with pregnancy termination. Our study contributes towards the discussion on unmet/met need for contraception and pregnancy termination across SSA. Governments of SSA and non-governmental organisations need to take pragmatic steps to increase met needs for contraception and also utilise mass media to encourage women to adhere to the prescription of contraceptives in order to reduce the incidence of unplanned pregnancies and unsafe abortions.

## Background

Sub-Saharan Africa (SSA) bears the highest burden of global reproductive ill-health with unsafe abortion being one of the most neglected aspects. Global projections indicate that 35 per 1,000 women terminated a pregnancy between 2010 and 2014, denoting 25% of all pregnancies worldwide and Africa accounted for 8.3% of all pregnancies terminated [1]. High burden of unintended pregnancies in lower-and middle-income countries (LMICs) occur partly as a result of the unmet need for contraception [2]. Over 75% of pregnancies terminated in Africa were unsafe [3] and 4.7%–13.2% of maternal deaths globally are attributable to unsafe pregnancy termination [4]. In conjunction with these statistics is the high cost of pregnancy termination borne by the ailing economies of SSA countries. For instance, US$ 553 million is spent every year to treat complications emerging from unsafe termination of pregnancies [5].

Over 26 years ago, 179 countries including those in SSA pledged to end unsafe pregnancy termination by ensuring that whenever the practice is legalised, it is conducted safely; safeguarding availability and easy access to family planning services; and fostering quality services for managing complications arising from pregnancy termination [6, 7]. However, due to stigma and restrictive laws across most SSA countries, pregnancy termination in the sub-region is mostly clandestine and unsafe thereby posing a major public health threat to women in the reproductive age [2, 8]. The poor, illiterates, and rural residents are the worst affected in SSA as far as complications of unsafe pregnancy termination are concerned [9].

To circumvent the adverse consequences, evidence highlight the need for effective contraceptive use [2, 10, 11]. However, unmet need for contraception is highest in SSA (23.4%) and exceeds the global average of 11.5%. This is only projected to decline to 20.3% by 2030 [12] with considerable inter-country disparities in contraceptive prevalence in the region [13]. SSA has the lowest demand for contraception globally (49.7%) which is far below the global average of 77.8% [13]. Women in SSA, therefore have a higher risk of experiencing pregnancy terminations, most of which may be unsafe with associated complications [9]. Unmet need for contraception is the proportion of women that want to stop/delay birth but not utilising contraception to prevent pregnancy and include pregnant women whose pregnancies are unplanned/mistimed when they became pregnant, as well as postpartum amenorrhoeic women who are not using family planning and whose last birth was unplanned/mistimed [14].

Hitherto, the few empirical studies that focused on the relationship between unmet need for contraception and pregnancy termination in SSA have been limited to some specific countries such as Ghana [15] and Ethiopia [16] or relied on old datasets [11, 17]. Evidence from these studies converge that the interaction between unmet/met needs for contraception is complex. This study extends the frontiers of evidence on the relationship between contraception needs and occurrence of pregnancy termination in SSA, where a high proportion of pregnancies terminated are unsafe [2, 18].

## Materials and methods

### Sources of data

This study used the most recent DHS data from 32 countries in SSA that were conducted between January 2010 and December 2018. Specifically, data was extracted from the women’s files of the DHS data sets of the countries. The DHS are national surveys carried out every five years in over 90 low- and middle- income countries globally [19]. The DHS concentrates on non-communicable diseases, maternal and child health issues, physical activity, sexually transmitted infections, fertility, health insurance, tobacco use, and alcohol consumption. The surveys mainly provide data to monitor the demographic and health profiles of the respective countries [19]. The sample for the present study consisted of women with unmet/met need for contraceptives (aged 15–49) and had complete cases on all variables of interest (N = 265,505). The DHS program granted us access to the dataset after the evaluation of our concept note. The datasets are freely available to the public at www.measuredhs.com.

### Study variables

#### Outcome variables

The outcome variable of this study was ever terminated a pregnancy. This was derived from the question “have you ever had a pregnancy terminated?”. It was coded as 0 = “No” and 1 = “Yes”. Undeniably, due to the measurement approach, this variable may include some spontaneous abortion cases. However, the range of induced abortion found in this study (9.13%–38.68%) and the average (16.27%) are comparable to the prevalence reported from some of the countries included in this study such as Burkina Faso (12%) [20], Nigeria (23%) [21], Ghana (24%–25%) [15, 22] and Ethiopia (33.6%) [23]. This shows that the majority of the reported prevalence in this study are induced abortions and as such findings and recommendations from the study may be instructive to governments of sub-Saharan Africa.

### Explanatory variables

The main explanatory variable was unmet/met need for contraception and thirteen other explanatory variables were considered as well. All these variables were grouped into individual and contextual level variables based on the hierarchical nature of the dataset. The variables were selected based on their availability in the dataset, practical significance and theoretical relevance for unmet/met need for contraception and pregnancy termination in previous studies [15, 24, 25].

### Individual level

Unmet/met need for contraception was accompanied by these responses: never had sex, unmet need for spacing, unmet need for limiting, no unmet need, not married and no sex in the last 30 days, and infecund and menopausal. Women who had never had sex, and infecund/menopausal women were excluded from the analysis because they were not exposed to the contraceptive need measurement [15, 25]. We then generated a binary measure of contraception needs by coding the rest of the responses into ‘unmet need’ (unmet need for spacing and unmet need for limiting) = 0 and ‘met need’ (no unmet need, using for spacing and using for limiting) = 1 [25]. The other explanatory variables were age, wealth status, education, marital status, and parity. Age was recorded as 15–19, 20–24, 25–29, 30–34, 35–39, 40–44, and 45–49. Wealth status was categorized into poorest, poorer, middle, richer, and richest. Education was classified into four categories: no education, primary education, secondary education, and higher education.

### Community level

Three variables were considered at the contextual level, namely place of residence, socio-economic disadvantage, and sex of head of household. The socio-economic disadvantage variable was generated from the education and occupation variables and captured as tertile 1(least disadvantaged), tertile 2 (moderate disadvantaged), and tertile 3 (most disadvantaged). The sex of the household head was captured as male and female.

### Statistical analysis

We employed both descriptive and inferential analytical approaches. First, we computed the proportion of women who had ever terminated a pregnancy (see Table 1). Following the hierarchical nature of the data set, the Multilevel Logistic Regression Model (MLRM) was employed. This comprises fixed effects, and random effects [26]. The fixed effects/measures of associations of the model were gauged with binary logistic regression which resulted in odds ratios (ORs) and adjusted odds ratios (aORs) (see Table 2). The random-effects/ measures of variations, on the other hand, were assessed with Intra-Cluster Correlation (ICC) [27] (see Table 2). All the analyses were carried out using STATA version 13.0.

**Table 1:** Background characteristics and proportion ever terminated pregnancy

Variables	Yes n (%)	Total n
Individual level		
Unmet/met need for contraception		
Unmet need	15	74,584
Met need	16	190,921
Age		
15–19	6	29,207
20–24	10	55,129
25–29	15	59,644
30–34	18	49,175
35–39	21	38,685
40–44	24	22,974
45–49	25	10,691
Level of education		
No education	14	91,758
Primary	17	86,903
Secondary	16	74,997
Higher	17	11,847
Marital status		
Single	8	31,155
Married	15	176,358
Cohabitation	21	43,548
Widowed	18	2,750
Separated	20	11,694
Parity		
Zero birth	11	30,494
One birth	12	44,141
Two births	15	43,479
Three births	16	38,021
Four or more births	19	109,370
Wealth status		
Poorest	15	56,782
Poorer	16	53,050
Middle	15	51,546
Richer	16	51,410
Richest	17	52,717
Community level		
Place of residence		
Urban	17	94,643
Rural	15	170,862
Sex of household head		
Male	16	204,517
Female	15	60,988
Socio-economic disadvantage		
Tertile 1 (Least disadvantage)	16	88,828
Tertile 2	16	88,301
Tertile 3 (Most disadvantage)	16	88,376

**Table 2:** Multilevel binary logistic regression results on the predictors of pregnancy termination among women with unmet/met need for contraception in sub-Saharan Africa

Variables	Model 0	Model 1 OR (95% CI)	Model 2 OR (95% CI)	Model 3 AOR (95% CI)
Individual level				
Unmet need for contraception				
Unmet need		1		1
Met need		1.11*** (1.07, 1.12)		1.11*** (1.07, 1.12)
Age				
15–19		1		1
20–24		1.87*** (1.76, 1.98)		1.87*** (1.77, 1.98)
25–29		2.86*** (2.69, 3.03)		2.87*** (2.71, 3.04)
30–34		3.77*** (3.54, 4.01)		3.79*** (3.55, 4.03)
35–39		4.73*** (4.43, 5.05)		4.76*** (4.46, 5.08)
40–44		5.74*** (5.36, 6.14)		5.78*** (5.39, 6.18)
45–49		6.17*** (5.72, 6.65)		6.22*** (5.77, 6.71)
Level of education				
No education		1		1
Primary		1.25*** (1.21, 1.28)		1.25*** (1.22, 1.29)
Secondary		1.32*** (1.28, 1.36)		1.33*** (1.28, 1.37)
Higher		1.13 (1.07, 1.20)		1.14 (1.08, 1.21)
Marital status				
Single		1		1
Married		1.68*** (1.60, 1.77)		1.66*** (1.58, 1.75)
Cohabitation		2.58*** (2.45, 2.72)		2.53*** (2.39, 2.67)
Widowed		1.45*** (1.31, 1.62)		1.49*** (1.34, 1.67)
Separated		2.14*** (2.00, 2.31)		2.17*** (2.03, 2.32)
Parity				
Zero birth		1		1
One birth		0.75*** (0.71, 0.79)		0.75*** (0.71, 0.79)
Two births		0.69*** (0.65, 0.72)		0.69*** (0.65, 0.72)
Three births		0.63*** (0.61, 0.67)		0.63*** (0.61, 0.67)
Four or more births		0.62*** (0.59, 0.66)		0.62*** (0.59, 0.66)
Wealth status				
Poorest		1		1
Poorer		1.02 (0.99, 1.06)		1.02 (0.99, 1.05)
Middle		0.97*** (0.94, 1.01)		0.97*** (0.93, 0.99)
Richer		0.98*** (0.95, 1.01)		0.95*** (0.92, 0.99)
Richest		0.99*** (0.96, 1.03)		0.94*** (0.90, 0.98)
Community level				
Place of residence				
Urban			1	1
Rural			0.89*** (0.86, 0.91)	0.92*** (0.91, 0.95)
Sex of household head				
Male			1	1
Female			0.93*** (0.91, 0.95)	0.93*** (0.91, 0.96)
Socio-economic disadvantage				
Tertile 1 (Least disadvantage)			1	1
Tertile 2			1.05* (1.01, 1.09)	1.10*** (1.06, 1.15)
Tertile 3 (Most disadvantage)			1.03 (0.99, 1.07)	1.09*** (1.05, 1.14)
Random effect result				
PSU variance (95% CI)	0.02 (0.1, 0.02)	0.02 (0.02, 0.03)	0.02 (0.01, 0.02)	0.02 (0.02, 0.03)
ICC	0.57	0.71	0.58	0.71
LR Test	χ^2^ = 158.11 p = 0.0000	χ^2^ = 196.68 p = 0.0000	χ^2^ = 157.85 p = 0.0000	χ^2^ = 200.96 p = 0.0000
Wald chi-square		7504.43	135.42	7584.83
Model fitness				
Log-likelihood	−114,738.7	−110,515.6	−114,671.1	−110,472.8
BIC	229502.3	221330.9	229417.1	221295.2
AIC	229481.3	221079.1	229354.2	221295.5
N	265,505	265,505	265,505	265,505

^*^p < 0.05 **p < 0.01 *** p < 0.001

### Model fit and specifications

We assessed the fitness of all the models with the Likelihood Ratio (LR) test. The presence of multicollinearity between the independent variables was checked before fitting the models. The variance inflation factor (VIF) test revealed the absence of high multicollinearity between the variables (Mean VIF = 2.98).

## Results

### Descriptive results

**Fig. 1:**
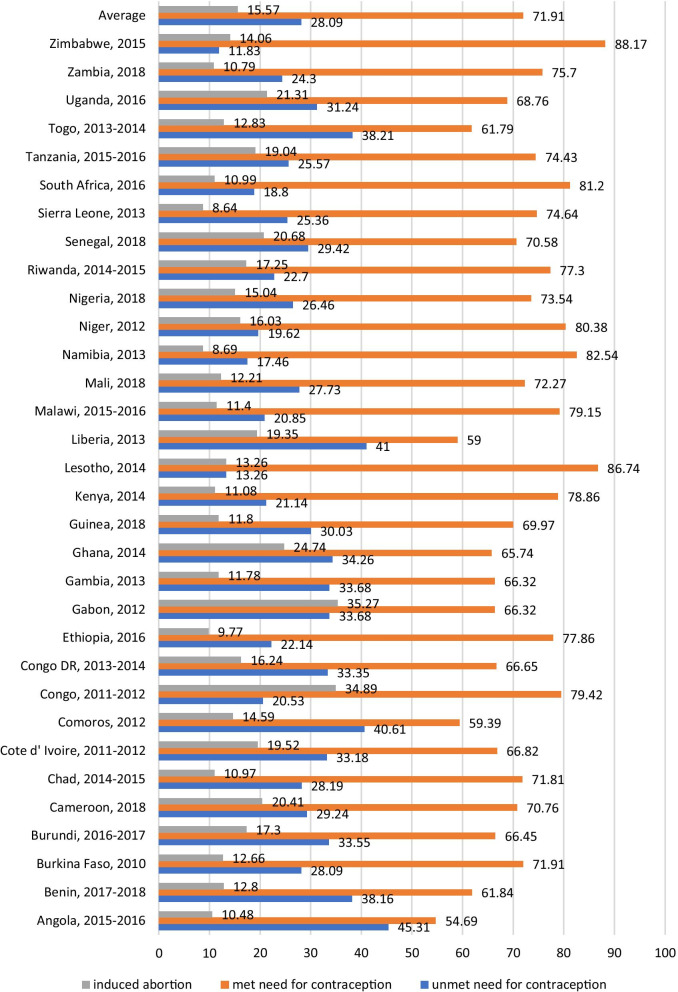
Proportion ever induced pregnancy

Figure 1 shows the proportion of women with unmet/met need for contraception that has ever terminated a pregnancy per country. On average, 28% of women in SSA have an unmet need for contraception. With regards to pregnancy termination, an average of 16% of women in SSA with unmet/met need for contraception had ever terminated a pregnancy, with Sierra Leone, recording the lowest proportion of 9% while Gabon had the highest proportion of 35%.

Table 1 shows a summary of the explanatory variables and the proportion of women who had ever terminated a pregnancy. Pregnancy termination was higher among women with a met need for contraception (16%), aged 45–49 (25%), those with primary education (17%), cohabiting women (21%), and those with four or more births (19%). Similarly, a greater section of women within the richest wealth quintile (17%), had terminated pregnancies. At the community level, women who reside in urban areas (17%), those with a male household head (16%) and those in moderately disadvantaged socio-economic status (16%) had a higher proportion of women who reported ever terminating a pregnancy.

### Fixed effects (measures of associations) results

In Table 2, Model 3 is the complete model showing the association between the individual level, contextual level, and pregnancy termination among women in SSA. At the individual level, unmet need for contraception, age, education, marital status, parity, and wealth index showed significant associations with pregnancy termination. At the community level, place of residence, sex of household head, and socio-economic disadvantage showed significant association with pregnancy termination.

The likelihood of terminating a pregnancy was higher for women with a met need for contraception [aOR = 1.11; 95% CI 1.07–1.12], as compared to their counterparts with unmet needs for contraception. Age was a strong factor in pregnancy termination with every additional age increasing the likelihood of pregnancy termination. Using no education as a reference, the likelihood of terminating a pregnancy increased with secondary education [aOR = 1.33; 95% CI 1.28–1.37]. Cohabiting women were more likely to terminate a pregnancy [aOR = 2.53; 95% CI 2.39–2.67], as compared to those who were single. Women with four or more children were found to be less likely to terminate a pregnancy [aOR = 0.62; 95% CI 0.59–0.66] as compared to those with zero parity. Women in the richest wealth quintile [aOR = 0.94; 95% CI 0.90–0.98] were less likely to terminate a pregnancy relative to poorest women.

At the community level, the likelihood of terminating a pregnancy was low for women having female household heads [aOR = 0.93; 95% CI 0.91–0.96] and women who reside in rural areas [aOR = 0.92; 95% CI 0.91–0.96]. On the contrary, moderately disadvantaged [aOR = 1.09; 95% CI 1.05–1.14], and most disadvantaged [aOR = 1.10; 95% CI 1.06–1.15] women were likely to terminate a pregnancy compared to least socio-economically disadvantaged women.

### Random effects (measures of variations) results

The empty model (Model 0) revealed minimal variation in the probability of pregnancy termination with respect to the clustering of PSUs (σ^2^ = 0.02, 95% CI 0.1, 0.02). The empty model further indicated that 57% of the overall variance in pregnancy termination is attributable to inter-cluster variation of the characteristics (ICC = 0.57). In the model 2, the probability of pregnancy termination did not vary (σ^2^ = 0.02, 95% CI 0.1, 0.02). However, there was a rise in the overall variance in pregnancy termination attributable to inter-cluster variation of the characteristics (71%). This indicates that the variation in pregnancy termination is highly attributable to differences or variations in factors at the community level as shown in Model 3.

## Discussion

This study investigates the relationship between unmet/met need for contraception and pregnancy termination in SSA. Unmet need for contraception, age, level of education, marital status, parity, wealth status, socio-economic disadvantage, and sex of household, had a significant association with pregnancy termination.

On average, 16% of women in SSA had ever terminated a pregnancy, with Sierra Leone, recording the lowest proportion of 9% while Gabon had the highest proportion of 39%. This is against the backdrop of Sierra Leone having a liberal abortion law that permits abortion under certain circumstances [28] while Gabon has a restrictive law on abortion [29]. This affirms the argument of Faundes and Shah [30] that women with unplanned pregnancy resort to abortion regardless of the laws, and countries with restrictive abortion laws record higher abortion rates. Hence, restrictive abortion laws may only force women to resort to unsafe abortion. We also found that women with a met need for contraception were more likely to terminate pregnancy compared to those who had an unmet need for contraception. This is consistent with a previous study by Amo-Adjei and Darteh [15] who reported that women with no unmet need for contraceptives had the highest odds of self-reported abortion in Ghana. A probable explanation of this is that contraceptives have a low failure risk [31] and this can translate into a high cumulative risk of unplanned pregnancies in a lifetime. There is therefore the chance of women being reluctant to utilise contraceptives due to previous failures [1]. Women with a met need for contraception may not necessarily be adhering to the prescription of contraceptives, hence increasing the risk of unplanned pregnancies. It is also worth mentioning that women with a met need for contraception might be relying on traditional methods which have a higher failure rate as compared to the modern methods [32]. This notwithstanding, the high rate of abortion among women with a met need for contraception could be attributed to women adopting post-abortion contraception [33].

We found that as the age of women increased, the odds of pregnancy termination proliferated as reported by earlier studies [34–36]. This may be due to longer exposure to unprotected sexual intercourse and the failure of traditional contraceptives such as the rhythm and calendar methods. Evidence has shown that most older women do not use modern contraceptives and a failure of the traditional methods may lead to the termination of pregnancy [37]. A probable explanation is that older women have a complete family and consequently are more prone to medical termination of unplanned pregnancies.

Women with secondary education had higher odds of pregnancy termination compared with those with no formal education. This is consistent with earlier studies by Yaya et al. [38] and Chae et al. [39]. Women with secondary education are more probable to be exposed to the knowledge of abortion services and may know locations or places where termination of pregnancy could be carried out. They may also be enlightened about the dangers of complications and the need to have a safe abortion.

Our study found a significant association between parity and pregnancy termination. Women with one birth and more were seen to have a lesser likelihood of pregnancy termination. Similar results were found in previous studies from Ghana and Mozambique [36, 40]. A possible explanation for this is that women in SSA are increasingly appreciating the importance of small family size [41]. We also found that women who had a female head of household reported a lower likelihood of pregnancy termination. This is inconsistent with the study of Izugbara [42]. This could be attributed to the fact that evidence on parent–child connectedness and or communication increasingly shows that women find it easier to discuss their sexual and reproductive health right (SRHR) issues with mothers or female guardians more than fathers or male guardians [43, 44].

Most socio-economically disadvantaged women had a higher likelihood of pregnancy termination. The possible explanation may be that possibly the most socio-economic disadvantaged may not have the means or wealth to take care of a baby. They may not be able to provide the nutritional and housing needs of the baby and hence the decision to terminate the pregnancy. It is possible that most socio-economically disadvantaged women may not want their children to experience the socio-economic hardship they are going through and hence the decision to terminate their pregnancies. Further, most socio-economically disadvantaged women may not have the financial capacity to afford a modern contraceptive and may be relying on traditional methods that have a higher failure rate [45].

## Strengths and limitations

This study employs a rigorous analytical approach in investigating the underlying factors predicting pregnancy termination in SSA. We used large, representative datasets of countries in SSA and these strengthen the validity and generalisability of our findings. These notwithstanding, the study had some shortcomings. First, the cross-sectional design of the study did not allow causal inference between the predictors and pregnancy termination. Second, depending on the social and neighbourhood factors of the women, there is a possibility of social desirability bias in their responses.

## Conclusion

The study has revealed that pregnancy termination persists among women in their reproductive age in SSA. Besides, women with a met need for contraception have higher odds of terminating a pregnancy. The underlying cause of this we argued could be poor adherence to the protocols of contraceptives or the reluctance of women to utilise contraceptives after experiencing a failure. This notwithstanding, pragmatic steps need to be taken to address the socio-economic disparities to promote the reproductive health and well-being of women. There is a need for efforts to intensify education on contraceptives and encourage adherence among women in their reproductive ages. Broader contextual factors need to be prioritised in the development of interventions aimed at mitigating pregnancy termination in SSA.

## Data Availability

Data used for the study is freely available to the public via https://dhsprogram.com/data/available-datasets.cfm.

## Abbreviations

AIC: Akaike Information Criterion
BIC: Bayesian Information Criterion
DHS: Demographic and Health Survey
ICC: Intra-Cluster Correlation
LMICs: Lower-and middle-income countries
LR: Likelihood Ratio
MLRM: Multilevel Logistic Regression Model
PSU: Primary Sampling Unit
SRHR: Sexual and Reproductive Health Right
SSA: Sub-Saharan Africa
